# Associations of potentially functional variants in *IL-6*, *JAKs* and *STAT3* with gastric cancer risk in an eastern Chinese population

**DOI:** 10.18632/oncotarget.8492

**Published:** 2016-03-30

**Authors:** Fei Zhou, Lei Cheng, Li-Xin Qiu, Meng-Yun Wang, Jin Li, Meng-Hong Sun, Ya-Jun Yang, Jiu-Cun Wang, Li Jin, Ya-Nong Wang, Qing-Yi Wei

**Affiliations:** ^1^ Cancer Institute, Collaborative Innovation Center for Cancer Medicine, Fudan University Shanghai Cancer Center, Shanghai 200032, China; ^2^ Department of Medical Oncology, Fudan University Shanghai Cancer Center, Department of Oncology, Shanghai Medical College, Fudan University, Shanghai 200032, China; ^3^ Department of Oncology, Shanghai Jiaotong University Affiliated Shanghai First People's Hospital, Shanghai 20080, China; ^4^ Department of Pathology, Fudan University Shanghai Cancer Center, Shanghai 200032, China; ^5^ Ministry of Education Key Laboratory of Contemporary Anthropology and State Key Laboratory of Genetic Engineering, School of Life Sciences, Fudan University, Shanghai 200433, China; ^6^ Fudan-Taizhou Institute of Health Sciences, Taizhou, Jiangsu 225300, China; ^7^ Department of Gastric Cancer & Soft Tissue Sarcoma Surgery, Fudan University Shanghai Cancer Center, Shanghai 200032, China; ^8^ Duke Cancer Institute, Duke University Medical Center, Durham, NC 27710, USA

**Keywords:** interleukin-6/JAK/STAT3, signaling pathway, genetic variate, gastric cancer, genetic susceptibility

## Abstract

The interleukin-6 (IL-6)/JAK/STAT3 signaling pathway plays a central role in inflammation-mediated cancers, including gastric cancer (GCa). We evaluated associations between 10 potentially functional single nucleotide polymorphisms (SNPs) of four essential genes in the pathway and GCa risk in a study of 1,125 GCa cases and 1,221 cancer-free controls. We found that a significant higher GCa risk was associated with *IL-6* rs2069837G variant genotypes [adjusted odds ratios (OR) = 1.33; 95% confidence interval (CI) = 1.12-1.59 for AG + GG vs. AA)] and *JAK1* rs2230587A variant genotypes (adjusted OR = 1.20; 95% CI = 1.02-1.43 for GA + AA vs. GG). We also found that a significant decreased GCa risk was associated with *STAT3* rs1053004G variant genotypes (adjusted OR = 0.84; 95% CI = 0.71-0.99 for AG + GG vs. AA). The combined analysis of *IL-6* rs2069837G and *JAK1* rs2230587A variant risk genotypes revealed that individuals with one-or-two risk genotypes exhibited an increased risk for GCa (adjusted OR = 1.34; 95% CI = 1.13-1.59). Genotypes and mRNA expression correlation analysis using the data from the HapMap 3 database provided further support for the observed risk associations. Larger studies are warranted to validate these findings.

## INTRODUCTION

Although the incidence of gastric cancer (GCa) is declining in recent decades, it remains high in Eastern Asia, particularly in China, where GCa ranks the second most diagnosed common cancer with an incidence of 15.8% and an estimated 0.68 million of new GCa cases in 2015 [1]. The etiology of GCa involves a combination of environmental factors, such as cigarette smoking, high salt intake, low consumption of fresh fruits and vegetables and *Helicobacter pylori* (*H. pylori*) infection [2, 3], and genetic factors, such as germline mutations and variants [4]. For example, one of the most common causes of GCa is infection of *H. pylori* that has been categorized by the WHO as a Class I Carcinogen since 1994 [5, 6]. The risk associated with *H. pylori* infection varies with age, geographical area and ethnic populations, but overall only15-20% of the infected have developed gastric ulcer disease and less than 1% have eventually suffered from GCa [6], which suggests that genetic factors play an essential role in GCa development.

The interleukin-6 (IL-6)/JAK/STAT3 signaling pathway has recently been shown to have a central role in inflammation-mediated cancers, such as those of the liver and stomach [7, 8]. Chronic gastritis leads an elevated expression of pro-inflammatory factors, such as IL-6, IL-1B, IL-8 and TNFα [9]. As one of the cytokines, IL-6 binds to IL-6 receptor-α (IL-6Rα) on the cell surface and induces receptor conformational changes, triggering the formation of a signaling complex composed of a gp130 and IL-6-IL-6Rα [10]. These events result in activation of JAKs that link to a cytoplasmic domain of gp130, and the activated JAKs mediate phosphorylation of gp130, leading to the recruitment and activation of cytosolic signal STAT3; the latter is then translocated into the nucleus and promotes various cellular processes that are required for cancer development [7, 8]. Consequently, persistently activated STAT3 itself induces the expression of many cytokines, including IL-6, and the receptors for these cytokines further activate STAT3, thus forming autocrine and paracrine positive feedback loops and leading to the promotion and amplification of cancer inflammation and finally the development of cancer [7, 8].

IL-6, JAK1, JAK2, and STAT3 are considered the essential components of the IL-6/JAK/STAT3 signaling pathway involved in promoting tumorigenesis [11], and these genes were observed to be overexpressed in GCa patients. For example, studies found that IL-6 expression levels were higher and associated with tumor progression in GCa patients [12, 13]; an increased STAT3 expression level was also found to be inversely correlated with survival in GCa patients [14, 15]; and inhibition of JAK2 reduced the growth of GCa [16, 17]. Because of the critical role of these genes in the pathway, we hypothesized that genetic variants of these genes were associated with GCa risk. In fact, studies have reported associations between single nucleotide polymorphisms (SNPs) of *IL-6* and risk of GCa, but these studies had a relatively small sample size, with only one larger study of 439 cases and 1138 controls conducted in European descendants [18–22]. Other studies have reported associations of some SNPs of *STAT3* and *JAK2* with risk of GCa, but all with less than 300 GCa patients [23].

To further test the hypothesis that genetic variants of *IL-6, JAK1, JAK2,* and *STAT3* in the IL-6/JAK/STAT3 signaling pathway are associated with GCa risk, we conducted a large case-control study in an eastern Chinese Han population by genotyping 10 selected, potentially functional SNPs of these essential genes in the pathway.

## RESULTS

### Characteristics of the study population

The final analysis included 1,125 GCa cases and 1,121 controls of Han Chinese recruited from our ongoing molecular epidemiology of GCa [24]. The distribution of demographic characteristics of the study subjects are presented in Table 1. Because cases and controls of the present study were frequency matched by age and sex, there was no statistical difference in their frequency distributions. The mean age was 58.60 ± 11.36 years for the cases and 58.93 ± 12.05 years for the controls (*P* = 0.557), and 71.1% of the cases and 69.8% of the controls were men (*P* = 0.314). However, there were more smokers and drinkers in the controls than in the cases (*P* = 0.0001 and *P* = 0.008, respectively). Therefore, these variables were further adjusted for in subsequent multivariate logistic regression analyses. Of the cases, 305 (27.1%) were gastric cardia adenocarcinoma (GCA) and 820 (72.9%) were gastric non-cardia adenocarcinoma (GNCA).

**Table 1:** Frequency distribution of demographic characteristics of gastric cancer cases and cancer-free controls in an eastern Chinese population

Variables	Cases *N* (%)	Controls *N* (%)	*P*^a^
All subjects	1,125	1,221	
Age, year			0.492
Range	21-86	22-89	
Mean^b^	58.60 ± 11.36	58.93 ± 12.05	
≤ 50	234 (20.8)	273 (22.4)	
51-60	383 (34.1)	387 (31.7)	
61-70	339 (30.1)	379 (31.0)	
> 70	169 (15.0)	182 (14.9)	
Sex			0.314
Female	325 (28.9)	376 (30.2)	
Male	800 (71.1)	845 (69.8)	
Smoking status			< 0.001
Never	686 (61.0)	622 (50.9)	
Ever	439 (39.0)	599 (49.1)	
Drinking status			0.008
Never	859 (76.4)	873 (71.5)	
Ever	266 (23.6)	348 (28.5)	
Tumor site^c^			
GCA	305 (27.1)		
GNCA	820 (72.9)		

a Two-sided χ^2^ test for distributions between cases and controls

b Data are presented as mean ± SD

c GCA: gastric cardia adenocarcinoma, GNCA, gastric non-cardia adenocarcinoma.

### Associations of the selected SNPs in *IL-6, JAK1, JAK2* and *STAT3* with GCa risk

The basic information of the 10 selected common, potentially functional SNPs identified by SNPinfo (http://snpinfo.niehs.nih.gov/snpfunc.htm) are summarized in Table 2. In an additive genetic model, SNPs *IL-6* rs2096837, *JAK1* rs2230587, *JAK2* rs1887429 and rs6476933 and *STAT3* rs1053005 seemed to be associated with an increased GCa risk, while SNPs *IL-6* rs2096840, *JAK1* rs10889513, *JAK2* rs3808850, and *STAT3* rs1053004 and rs4796793 seemed to be associated with a decreased GCa risk (Table 2). Although all the observed genotype frequencies among the controls were consistent with the Hardy-Weinberg equilibrium (*P* > 0.05) (Table 3), the genotype distributions between the cases and controls were significantly different for *IL-6* rs2069837 (*P* = 0.002), *JAK1* rs2230587 (*P* = 0.040) and *STAT3* rs1053004 (*P* = 0.037) under a dominant model but not for other seven SNPs (Table 3). Compared with the *IL-6* rs2069837 AA genotype, the G variant genotypes were associated with an increased risk of GCa (adjusted OR = 1.35, 95% CI = 1.01-1.45 for AG and adjusted OR = 1.33, 95% CI = 1.12-1.59 for AG + GG) after adjustment for age, sex, smoking and drinking status. Besides, an increased GCa risk was associated with *JAK1* rs2230587 A variant genotypes (adjusted OR = 1.43, 95% CI = 1.04-1.96 for AA and adjusted OR = 1.20, 95% CI = 1.02-1.43 for GA + AA), compared with the GG genotype after the adjustment. However, the *STAT3* rs1053004 G variant genotypes were associated with a decreased risk of GCa (adjusted OR = 0.75, 95% CI = 0.58-0.98 for GG and adjusted OR = 0.84, 95% CI = 0.71-0.99 for AG + GG), compared with the AA genotype after the same adjustment (Table 3).

**Table 2:** The basic information of selected, potentially functional SNPs in *IL-6, JAK1, JAK2* and *STAT3* collected from online prediction tool SNPinfo

Gene	rs no.	Chromosome No.	Gene region	Allele change	TFBS	miRNA	Minor allele	Frequency in CHB	OR (95% CI)*
*IL-6*	rs2069837	7	intron	A/G	Yes	—	A	0.827	1.01 (0.79-1.29)
*IL-6*	rs2069840	7	intron	C/G	Yes	—	C	0.899	0.77 (0.47-1.27)
*JAK1*	rs10889513	1	5′ near gene	G/A	Yes	—	G	0.600	0.95 (0.85-1.07)
*JAK1*	rs2230587	1	coding region	G/A	—	—	G	0.767	1.14 (0.98-1.32)
*JAK2*	rs1887429	9	5′ near gene	G/T	Yes	—	G	0.863	1.10 (0.88-1.39)
*JAK2*	rs3808850	9	5′ near gene	T/A	Yes	—	T	0.433	0.99 (0.89-1.10)
*JAK2*	rs6476933	9	5′ near gene	C/T	Yes	—	C	0.696	1.02 (0.89-1.17)
*STAT3*	rs1053004	17	3′UTR	A/G	—	Yes	A	0.690	0.89 (0.79-1.00)
*STAT3*	rs1053005	17	3′UTR	T/C	—	Yes	T	0.744	1.10 (0.94-1.27)
*STAT3*	rs4796793	17	5′ near gene	C/G	Yes	—	C	0.679	0.95 (0.84-1.07)

TFBS: transcription factor binding sites; UTR: untranslated region; CHB: Chinese Beijing.

* Estimated from our study population by the additive genetic model without adjustment

**Table 3:** Logistic regression analysis of associations of selected SNPs in *IL-6, JAK1, JAK2* and *STAT3* with gastric cancer risk in an eastern Chinese population

Variants	Genotype	Cases (N = 1,125)	Controls (N = 1,221)	*P*^a^	Crude OR (95% CI)	*P*	Adjusted OR (95% CI)	*P*^b^
*IL-6* **rs2069837 HWE_0.371**	AA	739 (65.7)	873 (71.5)	0.002^c^	1.00		1.00	
	AG	354 (31.5)	314 (25.7)		1.33(1.11-1.60)	0.002	1.35 (1.01-1.45)	0.001
	GG	32 (2.8)	34 (2.8)		1.11 (0.68-1.82)	0.673	1.14 (0.69-1.87)	0.611
	AG+GG	386 (34.3)	348 (28.5)	0.002^d^	1.31 (1.10-1.56)	0.003	1.33 (1.12-1.59)	0.002
	AA+AG	1093 (97.2)	1187 (97.2)		1.00		1.00	
	GG	32 (2.8)	47 (3.85)	0.930^e^	1.02(0.63-1.67)	0.930	1.04 (0.64-1.71)	0.873
*IL-6* **rs2069840 HWE_0.140**	CC	987 (87.7)	1043 (85.4)	0.203^c^	1.00		1.00	
	CG	132 (11.7)	167 (13.7)		0.84 (0.65-1.07)	0.149	0.80 (0.63-1.03)	0.079
	GG	6 (0.5)	11 (0.90)		0.58(0.21-1.57)	0.280	0.57 (0.21-1.55)	0.270
	CG+GG	138 (12.3)	178 (14.6)	0.101^d^	0.82 (0.65-1.04)	0.102	0.79 (0.62-1.01)	0.052
	CC+CG	1119 (99.5)	1210 (99.1)		1.00		1.00	
	GG	6 (0.5)	11 (0.90)	0.294^e^	0.59 (0.22-1.60)	0.230	0.58 (0.21-1.60)	0.295
*JAK1* **rs10889513 HWE_0.103**	GG	381 (33.9)	441 (36.1)	0.264^c^	1.00		1.00	
	GA	598 (53.1)	608 (49.8)		1.14 (1.03-1.47)	0.153	1.15 (0.96-1.37)	0.140
	AA	146 (13.0)	172 (14.1)		0.98 (0.76-1.27)	0.895	0.97 (0.75-1.27)	0.844
	GA+AA	744 (66.1)	780 (63.9)	0.254^d^	1.10 (0.93-1.31)	0.254	1.11 (0.93-1.32)	0.247
	GG+GA	979 (87.0)	1049 (85.9)		1.00		1.00	
	AA	146 (13.0)	184 (14.1)	0.433^e^	0.91 (0.72-1.15)	0.434	0.90 (0.71-1.14)	0.383
*JAK1* **rs2230587 HWE_0.657**	GG	554 (49.2)	653 (53.5)	0.067^c^	1.00		1.00	
	GA	473 (42.0)	484 (39.6)		1.15 (0.97-1.37)	0.103	1.16 (0.98-1.37)	0.092
	AA	98 (8.7)	84 (6.9)		1.38 (1.01-1.88)	0.046	1.43 (1.04-1.96)	0.026
	GA+AA	571 (50.8)	568 (46.5)	0.040^d^	1.19 (1.01-1.39)	0.040	1.20 (1.02-1.43)	0.030
	GG+GA	1027 (91.3)	1137 (93.1)		1.00		1.00	
	AA	98 (8.7)	84 (6.9)	0.098^e^	1.29 (0.95-1.75)	0.098	0.34 (0.99-1.82)	0.061
*JAK2* **rs1887429 HWE_0.391**	GG	773 (68.7)	867 (71.0)	0.419^c^	1.00		1.00	
	GT	313 (27.8)	319 (26.1)		1.10 (0.92-1.32)	0.307	1.12 (0.93-1.34)	0.250
	TT	39 (3.5)	35 (2.9)		1.25 (0.78-1.99)	0.349	1.26 (0.79-2.02)	0.333
	GT+TT	352 (31.3)	354 (29.0)	0.226^d^	1.12 (0.94-1.33)	0.226	1.13 (0.95-1.35)	0.180
	GG+TG	1086 (96.5)	1186 (97.1)		1.00		1.00	
	TT	39 (3.5)	35 (2.9)	0.406^e^	1.22 (0.77-1.94)	0.406	1.23 (0.77-1.96)	0.396
*JAK2* **rs3808850 HWE_0.189**	TT	347 (30.8)	373 (30.6)	0.977^c^	1.00		1.00	
	TA	575 (50.1)	624 (51.0)		0.99 (0.82-1.19)	0.920	0.99 (0.82-1.19)	0.899
	AA	203 (18.0)	224 (18.4)		0.97 (0.77-1.24)	0.831	1.00 (0.78-1.27)	0.983
	TA+AA	778 (69.2)	848 (69.5)	0.877^d^	0.99 (0.83-1.18)	0.877	0.99 (0.83-1.18)	0.915
	TT+TA	922 (82.0)	997 (81.7)		1.00		1.00	
	AA	203 (18.0)	224 (18.3)	0.850^e^	0.98 (0.79-1.21)	0.850	1.01 (0.81-1.24)	0.963
*JAK2* **rs6476933 HWE_0.683**	CC	531 (47.2)	570 (46.7)	0.892^c^	1.00		1.00	
	CT	481 (42.8)	533 (43.7)		0.97 (0.82-1.15)	0.915	0.97 (0.82-1.16)	0.767
	TT	113 (10.0)	118 (9.6)		1.03 (0.77-1.37)	0.849	1.04 (0.78-1.39)	0.775
	CT+TT	594 (52.8)	651 (53.3)	0.802^d^	0.98 (0.83-1.15)	0.802	0.99 (0.84-1.16)	0.872
	CC+CT	1012 (90.0)	1103 (90.3)		1.00		1.00	
	TT	113 (10.0)	118 (9.7)	0.758^e^	1.04 (0.80-1.37)	0.757	1.06 (0.80-1.39)	0.698
*STAT3* **rs1053004 HWE_0.067**	AA	445 (39.6)	432 (35.4)	0.044^c^	1.00		1.00	
	AG	549 (48.8)	614 (50.3)		0.87 (0.73-1.03)	0.114	0.87 (0.73-1.04)	0.117
	GG	131 (11.6)	175 (15.3)		0.73 (0.56-0.95)	0.017	0.75 (0.58-0.98)	0.035
	AG+GG	680 (60.4)	789 (64.6)	0.037^d^	0.84 (0.71-0.99)	0.037	0.84 (0.71-0.99)	0.048
	AA+AG	994 (88.4)	1046 (85.9)		1.00		1.00	
	GG	131 (11.6)	175 (14.1)	0.053^e^	0.79 (0.62-1.01)	0.053	0.82 (0.64-1.04)	0.103
*STAT3* **rs1053005 HWE_0.138**	TT	525 (46.7)	610 (50.0)	0.209^c^	1.00		1.00	
	TC	502 (44.6)	521 (42.6)		1.12 (0.95-1.33)	0.191	1.14 (0.96-1.35)	0.148
	CC	98 (8.7)	90 (7.4)		1.27 (0.93-1.72)	0.136	1.29 (0.95-1.77)	0.108
	TC+CC	600 (53.3)	611(50.0)	0.111^d^	1.14 (0.97-1.34)	0.111	1.16 (0.98-1.37)	0.080
	TT+TC	1027 (91.3)	1131 (92.6)		1.00		1.00	
	CC	98 (8.7)	90 (7.4)	0.232^e^	1.20 (0.89-1.62)	0.232	1.22 (0.90-1.65)	0.203
*STAT3* **rs4796793 HWE_0.823**	CC	431 (38.3)	478 (39.2)	0.560^c^	1.00		1.00	
	CG	548 (48.7)	569 (46.6)		1.07 (0.90-1.27)	0.461	1.08 (0.90-1.29)	0.405
	GG	146 (13.0)	174 (16.3)		0.93 (0.72-1.20)	0.582	0.97 (0.75-1.26)	0.813
	CG+GG	694 (61.7)	743 (60.9)	0.678^d^	1.04 (0.88-1.22)	0.678	1.05 (0.89-1.25)	0.546
	CC+CG	979 (87.0)	1047 (85.8)		1.00		1.00	
	GG	146 (13.0)	174 (14.2)	0.370^e^	0.90 (0.71-1.14)	0.371	0.93 (0.73-1.18)	0.550

Abbreviation: SNP, single nucleotide polymorphism; CI, confidence interval; OR, odds ratio.

a Chi square test for genotype distributions between cases and controls.

b Adjustment without (crude) and with age, sex, smoking and drinking status in logistic regression models.

c For additive genetic models.

d For dominant genetic models.

e For recessive genetic models.

Since the *STAT3* rs1053004 G variant genotypes were a protective factor for GCa, we then combined of the other two risk genotypes SNPs of *IL6* rs2069837 and *JAK1* rs2230587 in a dominant model to evaluate their joint effect, and we found that those with an increasing number of the risk genotypes had a significantly increased GCa risk in a genotype-dose response manner (*P*_trend_= 0.0002), compared with those who had no risk genotypes after the adjustment (Table 4). We also dichotomized the number of combined risk genotypes into a low-risk group (patients with 0 risk genotypes) and a high-risk group (patients with one-or-two risk genotypes) for further combined analysis. Compared with the low-risk group, the high-risk group had an obviously increased GCa risk (adjusted OR= 1.34, 95% CI 1.13-1.59, and *P* = 0.001) (Table 4).

**Table 4:** Combined effects of risk genotypes of *IL-6* and *JAK1* SNPs on GCa risk in an eastern Chinese population

NRG*	Cases (%)	Controls (%)	*P*^a^	Crude OR (95% CI)	*P*^b^	Adjusted OR (95% CI)	*P*^b^
0	365 (32.4)	474 (38.9)	0.002	1.00		1.00	
1	563 (50.0)	578 (47.3)		1.26 (1.06-1.51)	0.010	1.27 (1.06-1.53)	0.009
2	197 (17.5)	169 (13.8)		1.51 (1.18-1.94)	0.001	1.56 (1.22-2.01)	0.001
Trend test				0.004		0.0002	
0	365 (32.4)	474 (38.8)	0.001	1.00		1.00	
1-2	760 (67.6)	747 (61.2)		1.32 (1.12-1.57)	0.001	1.34 (1.13-1.59)	0.001

a Chi-square test was used to calculate the genotype frequency distributions.

b Obtained in logistic regression models without (crude) and with adjustment for age, sex, smoking and drinking status.

* NRG: numbers of risk genotypes; The risk genotypes include rs2069837 AG/GG and rs2230587 GA/AA.

### Stratification analysis

We further evaluated associations between variant genotypes of the significant SNPs identified in a single locus analysis and GCa risk by subgroups of age, sex, smoking and drinking status (Table 5). We found that the protective effect associated with the *STAT3* rs1053004 variant AG/GG genotypes was more evident in those who were younger (adjusted OR = 0.67, 95% CI = 0.46-1.00, *P* = 0.048), female (adjusted OR = 0.68, 95% CI = 0.50-0.92, *P* = 0.014), never-smokers (adjusted OR = 0.73, 95% CI = 0.59-0.92, *P* = 0.007) compared with those with the AA genotype (Table 5).

**Table 5:** Stratification analysis for associations between selected and combined genotypes and gastric cancer risk in an eastern Chinese population

Variables	*STAT3* rs1053004 (cases/controls)		Crude OR (95% CI)	*P*^a^	Adjusted OR (95% CI)	*P*^a^	NRG* (cases/controls)		Crude OR (95% CI)	*P*^a^	Adjusted OR (95% CI)	*P*^a^
	AA	AG+GG					0 genotypes	1-2 genotypes				
Age												
≤ 59	216/194	362/421	0.77 (0.61-0.98)	0.035	0.67 (0.46-1.00)	0.048	206/252	411/408	1.20 (0.95-1.52)	0.132	1.21 (0.95-1.53)	0.122
> 59	229/238	318/368	0.90 (0.71-1.14)	0.371	0.95 (0.75-1.12)	0.687	159/222	349/339	1.45 (1.15-1.86)	0.002	1.43 (1.12-1.84)	0.005
Sex												
Females	150/137	175/239	0.67 (0.49-0.91)	0.009	0.68 (0.50-0.92)	0.014	119/146	206/230	1.10 (0.81-1.49)	0.547	1.10 (0.81-1.49)	0.557
Males	295/295	505/550	0.92 (0.75-1.12)	0.407	0.94 (0.76-1.15)	0.534	246/328	554/517	1.43 (1.17-1.75)	0.001	1.45 (1.18-1.78)	0.0004
Smoking status												
Never	295/222	395/400	0.74 (0.60-0.93)	0.001	0.73 (0.59-0.92)	0.007	232/254	458/364	1.36 (1.09-1.71)	0.007	1.33 (1.06-1.67)	0.013
Ever	150/210	285/389	1.02 (0.79-1.33)	0.848	1.02 (0.80-1.33)	0.859	133/220	302/379	1.32 (1.01-1.72)	0.040	1.30 (1.00-1.70)	0.049
Drinking status												
Never	352/324	507/549	0.85 (0.70-1.03)	0.100	0.84 (0.69-1.03)	0.088	284/333	575/540	1.25 (1.03-1.52)	0.027	1.25 (1.03-1.53)	0.029
Ever	93/108	173/240	0.84 (0.60-1.18)	0.304	0.86 (0.61-1.20)	0.366	81/220	302/379	1.56 (1.10-2.18)	0.010	1.55 (1.10-2.18)	0.011

* NRG: number of risk genotypes of *IL6* rs2069837AG/GG and *JAK1* rs2230587GA/GG.

a Obtained in logistic regression models without (crude) and with adjustment for age, sex, smoking and drinking status.

Then, we performed stratification analysis for risk associated with the combined risk genotypes of *IL-6* rs2069837 and *JAK1* rs2230587 and found that those who carried 1-2 risk genotypes had an increased risk, and the risk was more evident in those who were of older ages (adjusted OR = 1.43, 95% CI = 1.12-1.84, *P* = 0.005), male (adjusted OR = 1.45, 95% CI = 1.18-1.78, *P* = 0.0004), both smokers (adjusted OR = 1.30, 95% CI = 1.00-1.70, *P* = 0.049) and non-smokers (adjusted OR = 1.33, 95% CI = 1.06-1.67, *P* = 0.013), both drinkers (adjusted OR = 1.55, 95% CI = 1.10-2.18, *P* = 0.011) and non-drinkers (adjusted OR = 1.25, 95% CI = 1.03-1.53, *P*= 0.029), compared with those with 0 risk genotypes (Table 5).

### Expression quality trait loci (eQTL) analysis by *IL-6, JAK1* and *STAT3* genotypes in lymphoblastoid cell lines

**Figure 1:**
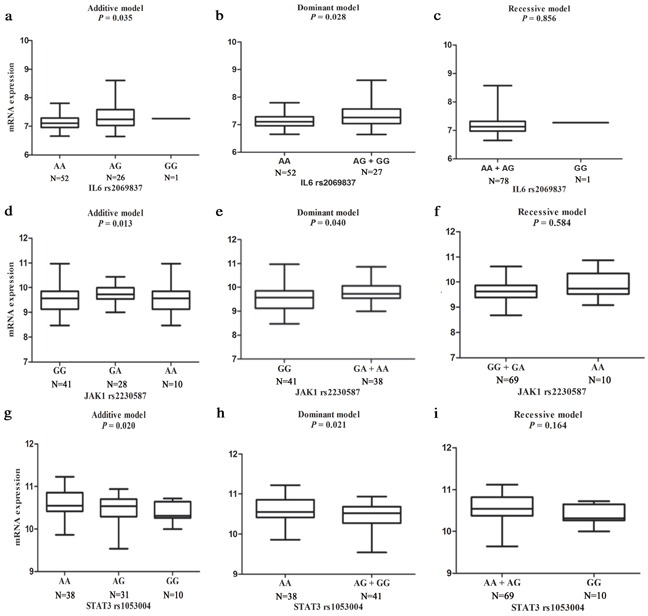
eQTL analysis of mRNA expression for genotype-phenotype correlation analysis in three different genetic models (additive, dominant and recessive) from EBV-transformed B lymphoblastoid cell lines of 79 unrelated Chinese people included in HapMap 3 database **a, b, c.** for *IL-6* rs2069837; **d, e, f.** for *JAK1* rs2230587; and **g, h, i.** for *STAT3* rs1053004.

Finally, we performed genotype-phenotype correlation analysis by using mRNA expression data of the lymphoblastoid cell lines in 79 unrelated Chinese people available in the HapMap 3 database to provide additional support for our findings. As shown in Figure 1a and 1b, with the increase in the number of allele, mRNA expression levels of *IL-6* was significantly increased in both additive (*P* = 0.035) and dominant (*P* = 0.028) models. For *JAK1*, the mRNA expression levels were also increased as the number of the rs2230587A allele increased, which is displayed in Figure 1d and 1e in both additive (*P* = 0.013) and dominant (*P* = 0.040) models. The *STAT3* rs1053004 G allele exhibited a protection against GCa risk, and the mRNA expression levels of *STAT3* were consistent with additive (*P* = 0.020) and dominant (*P* = 0.021) models in Figure 1g and 1h. The gene mRNA expressions of other non-significant, potentially functional SNPs were shown in the Supplementary Figures S1 (except for rs1800796, rs17097146, and rs1053023, which has no mRNA expression data in HapMap 3 database).

## DISCUSSION

To our knowledge, this was the first study that investigated whether the selected, potentially functional SNPs in four essential genes (i.e., *IL-6, JAK1, JAK2*, and *STAT3*) of the IL-6/JAK/STAT3 signaling pathway were associated with GCa risk in a large, ethnic-specific and single institutional case-control study. In the present study, we found that both the *IL-6* rs2069837 AG/GG and *JAK1* rs2230587 GA/AA were associated with an increased GCa risk, and the effects were more evident in subgroups of older age (> 59 year), males, never and ever-smokers, and never and ever-drinkers. We also found that *STAT3* rs1053004 was associated with a decreased GCa risk, and the risk was more obvious in those who were younger (≤ 59 year), females and never-smokers. Both the risk and protective effects associated with the SNPs appeared to be supported by their roles in modulating mRNA expression levels of their genes based on the HapMap 3 database for the unrelated Chinese people.

Located on Chromosome 7, the *IL-6* gene encodes a single-chain glycoprotein of 184 amino acid, mainly produced by both hematopoietic and epithelial cells, and regulates diverse inflammatory processes [7, 10, 25]. It is well known that inflammatory responses play important roles in cancer development, including tumor initiation, promotion and progression [26]. It was also reported that a persistent JAK-STAT3 activation by IL-6 induces a chronic inflammatory state and thus affects gastric epithelial cell turnover, leading to gastric tumorigenesis [27, 28].

In the present study, we found that *IL-6* rs2069837 AG/GG genotypes were associated with an increased GCa risk. To our knowledge, this is the first report that the *IL-6* rs2069837 SNP is associated with an increased GCa risk. To date, most published studies of associations between *IL-6* SNPs and cancer risk focused on rs1800795 and rs1800796. According to function prediction of the online tool SNPinfo, rs1800796 is in high LD (r^2^> 0.8) with rs2069837. Studies in Chinese populations have shown that the rs1800796 SNP was a risk factor for lung cancer in a meta-analysis [29] and that the rs2069837 SNP was a risk factor for cervical cancer [30]. In addition, the rs2069837 SNP seemed to be associated with an increased GCa risk in a Korea population with *H. pylori* infection, although it did not reach statistical significance, perhaps because the sample size was not large enough to detect a weak association [22]. In the present study, we found that the increasing of *IL6* mRNA expression was associated with the increasing number of the G allele in 79 unrelated Chinese people obtained from the HapMap 3 database, which is consistent with the phenomenon that IL-6 expression levels were higher in GCa patients than in health controls [12, 31].

*JAK1*, located on chromosome 1p31.3, encodes a protein tyrosine kinase that acts as an essential mediator protein in the IL-6/JAK/STAT3 pathway and transmits the signals from IL-6 to STAT3. This protein is involved in cell growth, survival, development, and differentiation of a variety of cells but are critically important for both immune and hematopoietic cells [32]. In animal experiments, mice with the *JAK1* deficiency died perinatally [33, 34].

In the present study, we also found that the *JAK1* rs2230587 SNP was associated with an increased GC risk in a dominant model, and elevated mRNA expression levels of the gene were also associated with the A variant genotypes. To date, no published studies have reported the association of rs2230587 with cancer risk. The SNP rs2230587 is located in the exon 3 of *JAK1*, and the substitute of G by A does not change the amino acid. However, as predicted by SNPinfo (http://snpinfo.niehs.nih.gov/snpinfo/snpfunc.htm), rs2230587 is on a splicing site that could have an impact on the mRNA expression. The change in the mRNA expression possibly leads to the change of protein composition, function and signal transduction in the IL-6/JAK/STAT3 signaling pathway, a possible mechanism that may lead to the susceptibility to GCa.

STAT3 is the pivotal essential component of the IL6/JAK/STAT3 signaling pathway. Located on chromosome 17q, *STAT3* has traditionally been recognized as an oncogene. It encodes a protein that is constitutively activated in a variety of human cancers, including GCa, and plays critical roles in cancer cell proliferation, survival, metastasis and angiogenesis [8, 14, 35].

We investigated three SNPs of the *STAT3* in the present study and found that the rs1053004 AG/GG genotypes were associated with a lower risk of GCa. To our knowledge, this finding is also the first to describe an association between rs1053004 and cancer risk. SNP rs1053004 is located in a predicted miRNA-binding site for miR-423-5p at the 3′UTR of *STAT3*, which may have an impact on the binding of the miRNA to mRNA and degradation of the mRNA. The HapMap3 data also revealed that the mRNA expression levels of *STAT3* was were decreased in the subjects with AG or GG genotypes, compared with those with the AA genotype.

In our present study, we did not find an association between *STAT3* rs4796793 and GCa risk. The rs4796793 SNP, together with rs744166 that is in high LD with rs4796793 (r^2^>0.8), is the most widely studied *STAT3* SNP in various cancers. However, two studies reported some opposite results in association with GCa risk; one Chinese study found that rs744166 SNP might be associated with a decreased risk of GCa in an Northeast Chinese population with 209 GCa patients and 294 cancer-free controls, while a Brazilian study showed that rs744166 SNP was associated with an increased GCa risk in a South American population with 232 GCa cases and 541 cancer-free controls [23, 36]. The inconsistent results of three studies (including the present study) may be explained by the differences in sample sizes and study populations with different exposures, such as age, smoking, and alcohol use, which is also consistent with the results of our stratified analysis, in which the risk varied among the subgroups whose samples were further reduced.

The present study showed the importance of simultaneously investigating four essential genes in a pathway, because the combination of multi-SNPs in the same pathway may reveal much stronger effects than any single SNP. Indeed, the significant combined effect of the risk genotypes of *IL-6* and *JAK1* was in an allele dose response manner, as the number of adverse genotypes increased.

In summary, the present study we investigated the associations between 10 selected, potentially functional SNPs of four essential genes involved in the IL-6/JAK/STAT3 pathway and GCa risk with a relatively large sample size. However, some limitations should be considered. First, although the subjects in the present study came from eastern China, it was a hospital-based case-control study that is subject to inherent selection biases by the non-representative subjects and retrospective collection of exposure data (e.g., lack of data on family history and *H Pylori* infection status). Second, the present study included more smokers and drinkers in the controls than in the cases, which may not be fully adjusted in the multivariate analysis. Finally, only 10 potentially functional SNPs in four genes were investigated, which did not cover all other functional SNPs in the pathway and may have missed some important, truly functional ones. Therefore, additional larger, well-designed population-based studies are warranted to confirm our findings, and further molecular functional assays are needed to explore the mechanisms for potentially functional SNPs in the IL-6/JAK/STAT3 pathway.

## MATERIALS AND METHODS

### Study subjects

The study population consisted of 1,130 unrelated Han Chinese patients with newly diagnosed and histopathologically confirmed primary gastric adenocarcinoma from an ongoing molecular epidemiology study [23, 24] at Fudan University Shanghai Cancer Center (FUSCC) between 2009 and 2011. All patients came from eastern China, including Shanghai, Jiangsu, Zhejiang, Anhui and the surrounding regions. In addition, 1,226 Han ethnic controls, who were frequency matched to the cases on age (5 years) and sex, were recruited from Taizhou Longitudinal Study (TZL) in an area of Jiangsu province that in the vicinity of Shanghai located in eastern China at the same time period with the selection criteria of no history of any cancer [37]. Blood samples of these GCa patients and cancer-free controls were provided by the tissue bank of FUSCC and the TZL study, respectively. A written informed consent was signed by all participants for donating their biological samples to the tissue bank for scientific research. This study was approved by the Institutional Review Board of FUSCC.

### SNP selection and genotyping

All possible, independent potentially functional SNPs of *IL-6, JAK1, JAK2,* and *STAT3* in the IL-6/JAK/STAT3 pathway were selected from the NCBI dbSNP database (http://www.ncbi.nlm.nih.gov/projects/SNP) and SNPinfo (http://snpinfo.niehs.nih.gov/snpfunc.htm) based on the following criteria: (1) located at the transcription factor binding site (TFBS) in the putative promoter region; (2) located at the microRNA (miRNA) binding site activity; (3) located at a splicing site; (4) the minor allele frequency (MAF) of at least 5 % in Chinese populations;, (5) with a low linkage disequilibrium (LD) with other SNPs using an r^2^ threshold of < 0.8 as the cut-off value for each other, and (6) not included in the published genome-wide association studies (GWASs). As a result, 10 selected SNPs and their functional relevance are summarized in Table 2. The SNPs tagged by these 10 selected SNPs and their functional relevance are also summarized in Supplementary Table S1. For the *IL-6* gene, we chose rs2069837 and rs2069840 that are both located in an intron but may affect the TFBS activity. For the *JAK1* gene, we chose rs10889513 and rs2230587; the former located in the 5′ near gene region may affect the TFBS activity, and the latter located in the coding region may affect the structure of protein. For the *JAK2* gene, we chose rs1887429, rs3808850, and rs6476933 that are all located in the 5′ near gene region and may affect the TFBS activity. For the *STAT3* gene, we chose rs1053004, rs1053005 and rs4796793; the former two located in the 3′UTR region may affect the miRNA binding site activity and the latter one located in the 5′ near gene region may affect the TFBS activity. All these 10 selected SNPs were genotyped by the Taqman real-time PCR method.

Genomic DNA extraction from blood samples and DNA genotyping were conducted as described previously [38], and the samples from 1,130 cases and 1,226 controls were genotyped using the Taqman assays, but 1,125 cases and 1,221 controls were successfully genotyped with a calling rate of 99.6%. The discrepancy rate in 10% of duplicated samples was less than 0.1%, and a few samples were also randomly selected to be sequenced to confirm the genotypes.

### Genotype and mRNA expression data of lymphoblastoid cell lines from the HapMap3 database

We also used additional data on genotypes of the four genes in the IL-6/JAK/STAT3 signaling pathway (http://hapmap.ncbi.nlm.nih.gov/downloads/genotypes/2010-05_phaseIII/) and mRNA expression levels available online (http://www.ebi.ac.uk/arrayexpress/experiments/E-MTAB-264/) for the genotype-phenotype association analysis. The genotyping data were from the HapMap phase 3 release 3 dataset consisting of about 1.6 million SNP genotypes of 692 individuals from 11 populations [39]. The mRNA expression data together with genotypes were derived from EBV-transformed B lymphoblastoid cell lines obtained from 726 individuals from eight global populations from the HapMap3 Project, from which data on 79 unrelated Chinese subjects were used for the genotype-phenotype correlation. Illumina's commercial whole genome expression array, Sentrix Human-6 Expression BeadChip version 2 was used to assay the mRNA expression levels, and eQTL analysis was employed to evaluate the genotype-phenotype correlation [40].

### Statistical analysis

Pearson's χ^2^ test was used to compare differences in the distributions of categorical variables, including selected demographic variables and other covariates, between the cases and controls. A goodness-of-fit χ^2^ test was employed to test the Hardy-Weinberg equilibrium of the control genotype distributions. Both univariate and multivariate logistic regression analyses with adjustment for age, sex, smoking and drinking were used to evaluate ORs and 95% CIs for the estimation of GCa risk. The association was also evaluated in subgroup analyses stratified by demographic and risk factors. Linear regression analysis was applied for the comparison of mRNA levels between samples of different genotypes. All tests were two-sided using the Statistical Analysis Software (v.9.2 SAS Institute, Cary, NC), and *P* < 0.05 was considered statistically significant.

## SUPPLEMENTARY FIGURES AND TABLES



## Abbreviations

CI: confidence interval
GCa: gastric cancer
GCA: gastric cardia adenocarcinoma
*H. pylori*: *Helicobacter pylori*
IL-6: interleukin
IL-6 receptor-α: IL-6Rα
LD: linkage disequilibrium
MAF: minor allele frequency
GNCA: gastric non-cardia adenocarcinoma
OR: odds ratios
SNP: single nucleotide polymorphism
TFBS: transcription factor binding site
