# Supplementary material for: Associations of potentially functional variants in IL-6, JAKs and STAT3 with gastric cancer risk in an eastern Chinese population
Source: Oncotarget. 2016 Mar 30;7(19):28112–23. doi: 10.18632/oncotarget.8492 (PMC5053713; doi:10.18632/oncotarget.8492)
Supplement: Supplementary file 1 [file oncotarget-07-28112-s001.pdf]

SUPPLEMENTARY FIGURES AND TABLE

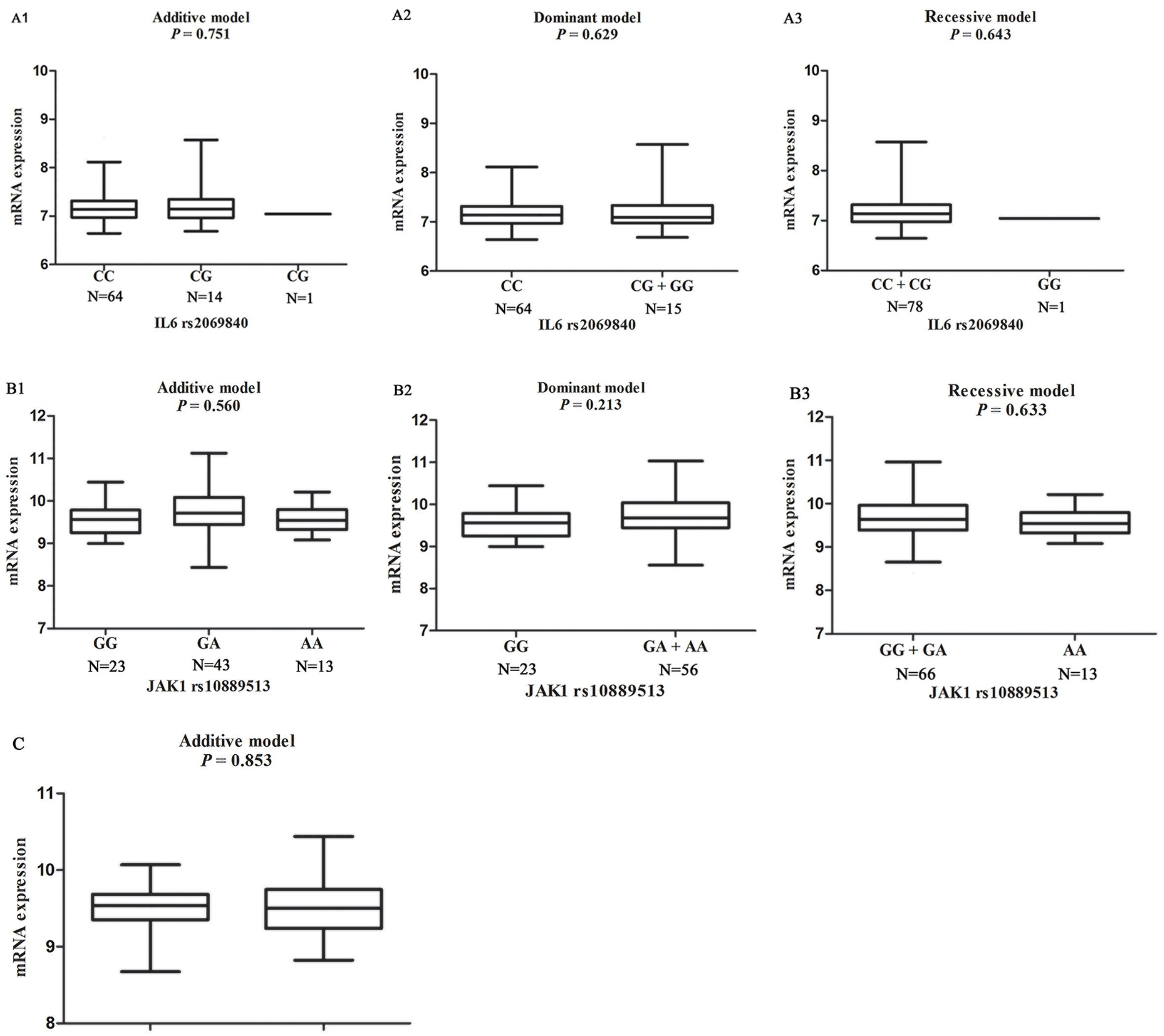

Supplementary Figure S1: eQTL analysis of mRNA expression for genotype-phenotype correlation analysis in three different genetic models (additive, dominant and recessive) from EBV-transformed B lymphoblastoid cell lines of 79 unrelated Chinese people included in HapMap 3 database. A1–A3. for *IL-6* rs2069840. **B1–B3.** for *JAK1* rs10889513. **C.** for *JAK1* rs1887429. (*Continued*).

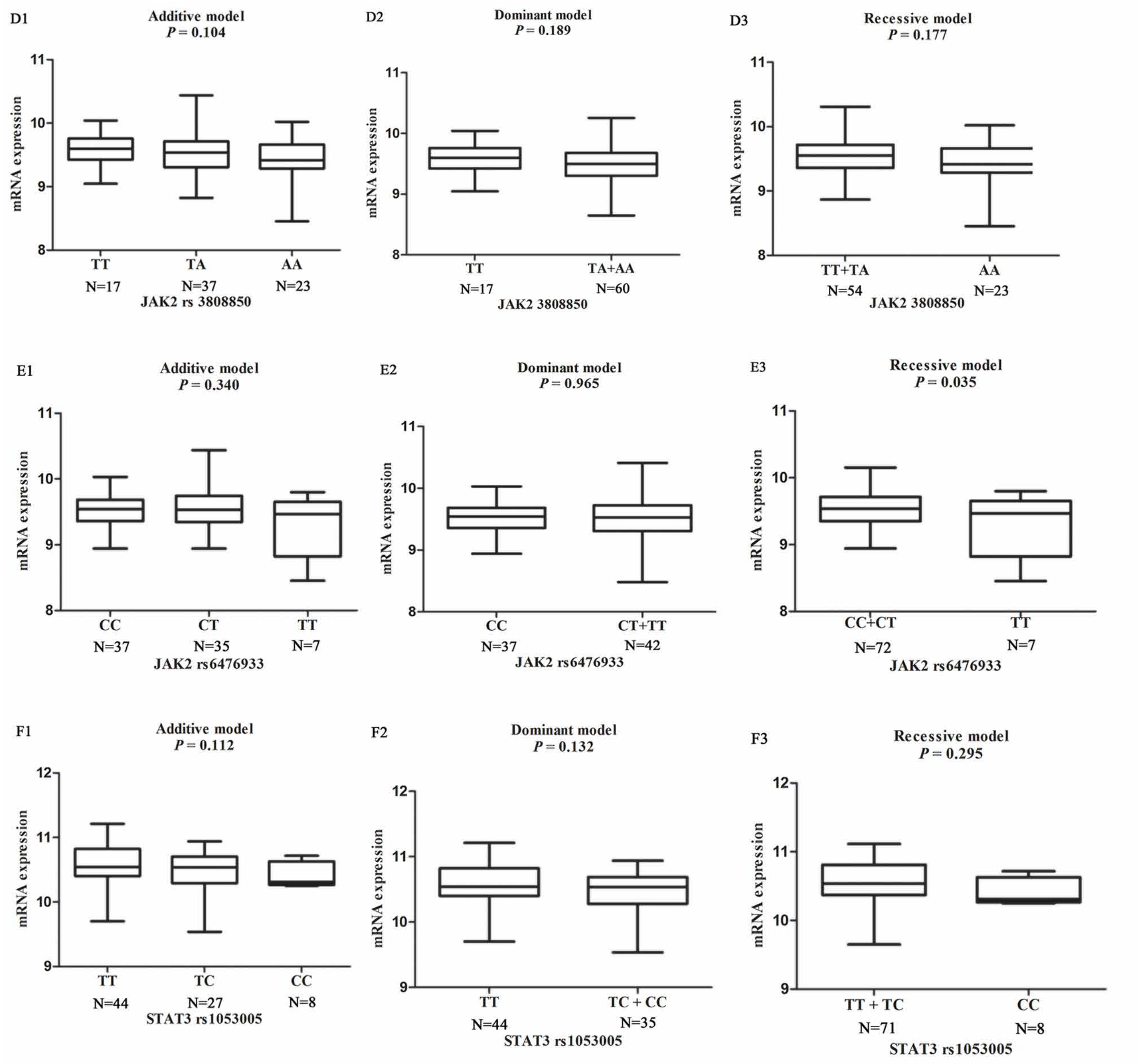

Supplementary Figure S1: (*Continued*) eQTL analysis of mRNA expression for genotype-phenotype correlation analysis in three different genetic models (additive, dominant and recessive) from EBV-transformed B lymphoblastoid cell lines of 79 unrelated Chinese people included in HapMap 3 database. D1–D3. for *JAK2* rs3808850. **E1–E3.** for *JAK2* rs6476933. **F1–F3.** for *STAT3* rs1053005. (*Continued*).

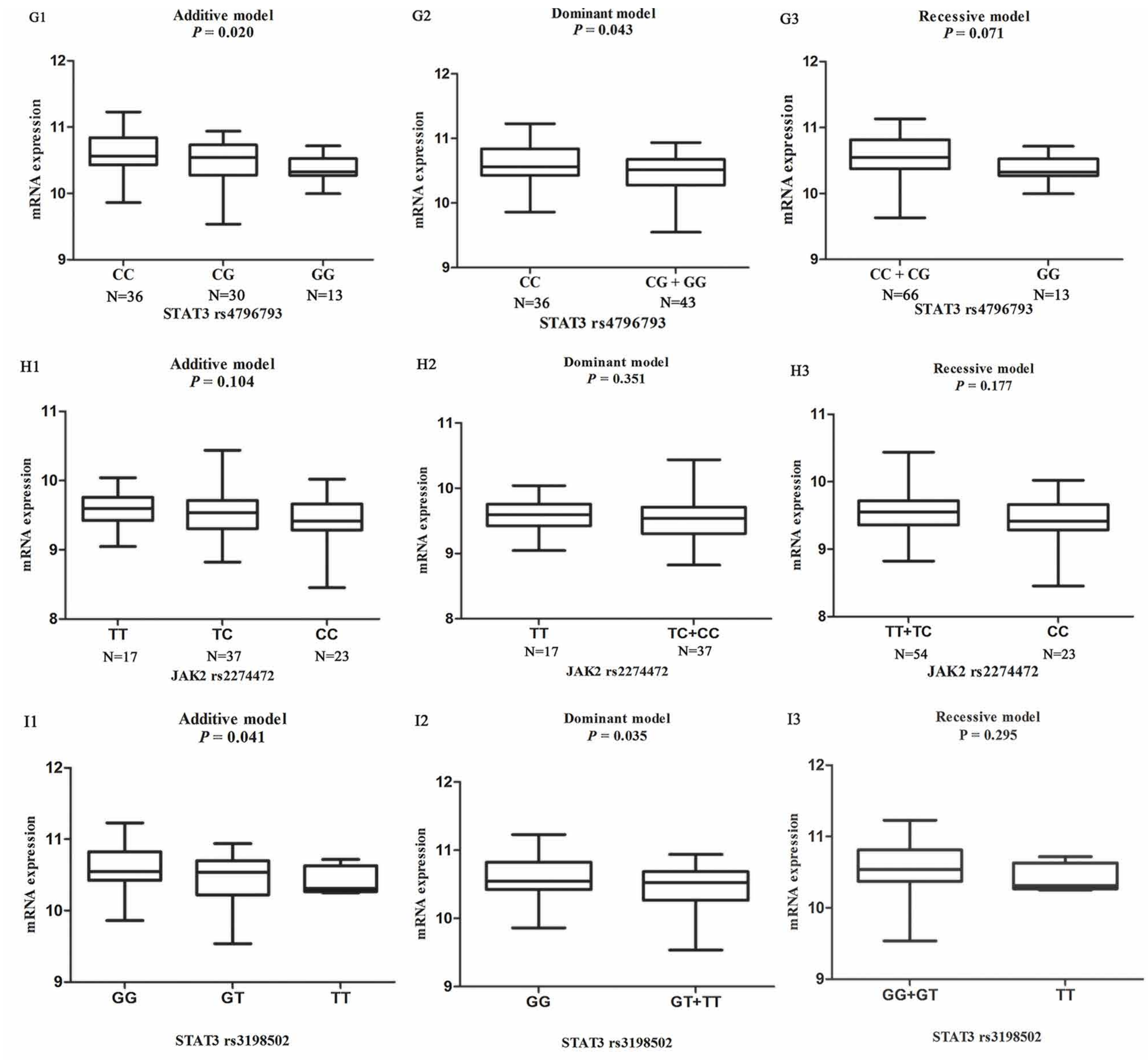

Supplementary Figure S1: (*Continued*) eQTL analysis of mRNA expression for genotype-phenotype correlation analysis in three different genetic models (additive, dominant and recessive) from EBV-transformed B lymphoblastoid cell lines of 79 unrelated Chinese people included in HapMap 3 database. G1–G3. for *STAT3* rs4796793. **H1–H3.** for *STAT3* rs2274472. **I1–I3.** for *STAT3* rs3198502. (*Continued*).

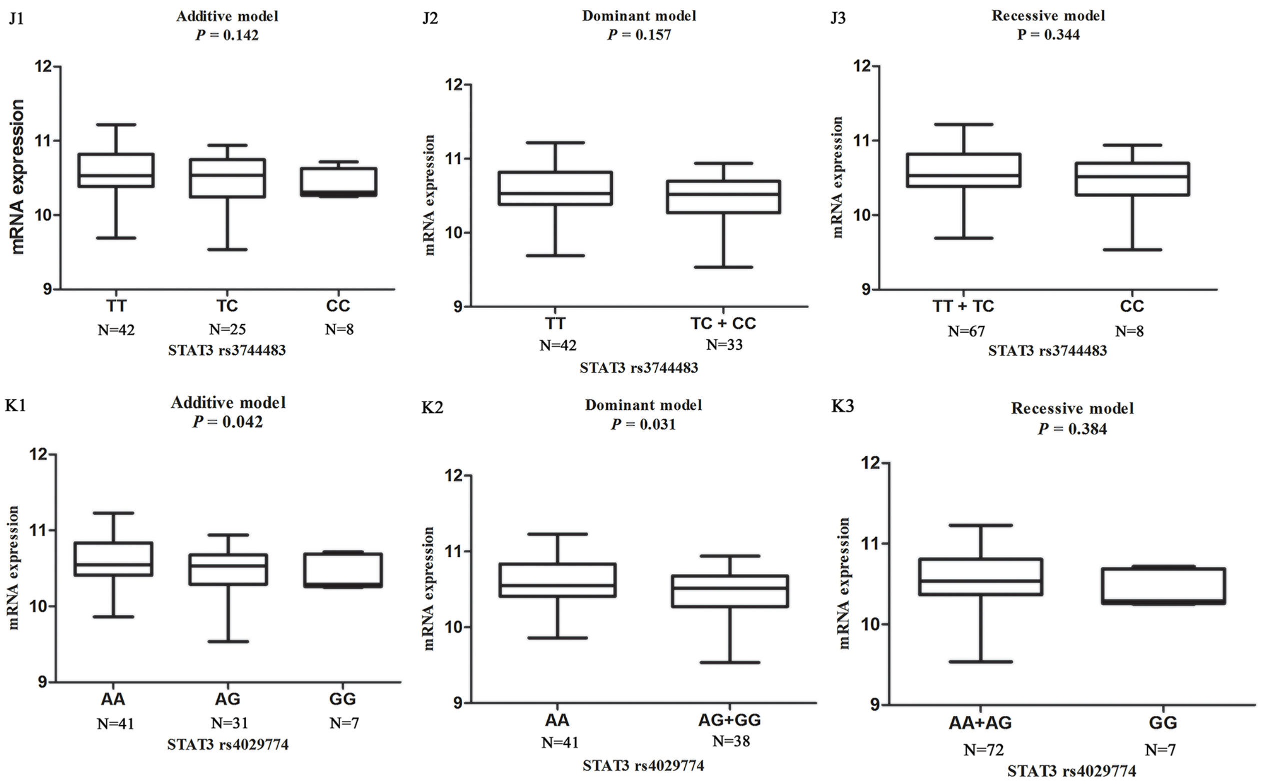

Supplementary Figure S1: (*Continued*) eQTL analysis of mRNA expression for genotype-phenotype correlation analysis in three different genetic models (additive, dominant and recessive) from EBV-transformed B lymphoblastoid cell lines of 79 unrelated Chinese people included in HapMap 3 database. J1–J3. for *STAT3* rs3744483. **K1–K3.** for *STAT3* rs4029774.

Supplementary Table S1: The ten selected and genotyped SNPs and those SNPs they tagged based on the online prediction tool SNPinfo and Hapmap 3 as validated in Chinese populations

Gene	SNPs selected and genotyped	SNPs in LD ($r^2 \geq 0.8$) or tagged but not genotyped
IL-6	rs2069837	rs11981074, rs1800796*
	rs2096840	rs12956846, rs12956909, rs206590
JAK1	rs10889513	rs12409333, rs12730021, rs1497056, rs4587600, rs4915675, rs7546535, rs7546545, rs7553101
	rs2230587	rs10493364, rs10889499, rs12406400, rs17097146*, rs17127090, rs17127107, rs17127117, rs1887050, rs2230587, rs2478151, rs3762311, rs3790541, rs4916008, rs6679426, rs914960;
JAK2	rs1887429	None
	rs3808850	rs2274472*
	rs6476933	None
STAT3	rs1053004	rs12601982, rs9895297
	rs1053005	rs1053023*, rs12601982, rs16967611, rs16967620, rs2272087, rs2293154, rs2293155, rs3198502*, rs34129849, rs3744483*, rs3809758, rs4029774*, rs6503692, rs7209222, rs7218653, rs8064638, rs8074524, rs8078731, rs8080122, rs8082188, rs8082391, rs9906933, rs9906989, rs9907247, rs9909659
	rs4796793	rs1026916, rs11079043, rs12942547, rs12949918, rs17885629, rs3785898, rs3816769, rs4103200, rs4796791, rs6503695, rs6503696, rs6503697, rs7211777, rs7217655, rs7219739, rs744166, rs8069645, rs957970, rs963988, rs9891119, rs9912773

*Also potentially functional as predicted by SNPinfo.
